# Supplementary material for: Fast Hadamard-Encoded 7T Spectroscopic Imaging of Human Brain
Source: Tomography. 2025 Jan 13;11(1):7. doi: 10.3390/tomography11010007 (PMC11769540; doi:10.3390/tomography11010007)
Supplement: Supplementary file 1 [file tomography-11-00007-s001.zip › tomography-3361838-supplementary.pdf]

Supplementary Material

Eight-slice Hadamard encoded single spin echo rosette spectroscopic imaging (RSI) acquisition: To demonstrate the multi-slice capability of the cascaded RF pulse encoded by Hadamard in slice direction, Figure S1 shows the magnitude images of NAA acquired with the eight-slice Hadamard RSI with 16 min scan duration using N_{sh} = 40; the slices are 4mm thick with a 1mm slice gap slice to span a 40 mm slab. TEs for the respective eight slices are 35, 42, 49, 56, 62, 70, 77 and 83 ms. More detail image acquisitions and reconstruction are described at Methods section of *2.1. Multi-slice Hadamard-encoded sequence: cascaded and simultaneous excitation*.

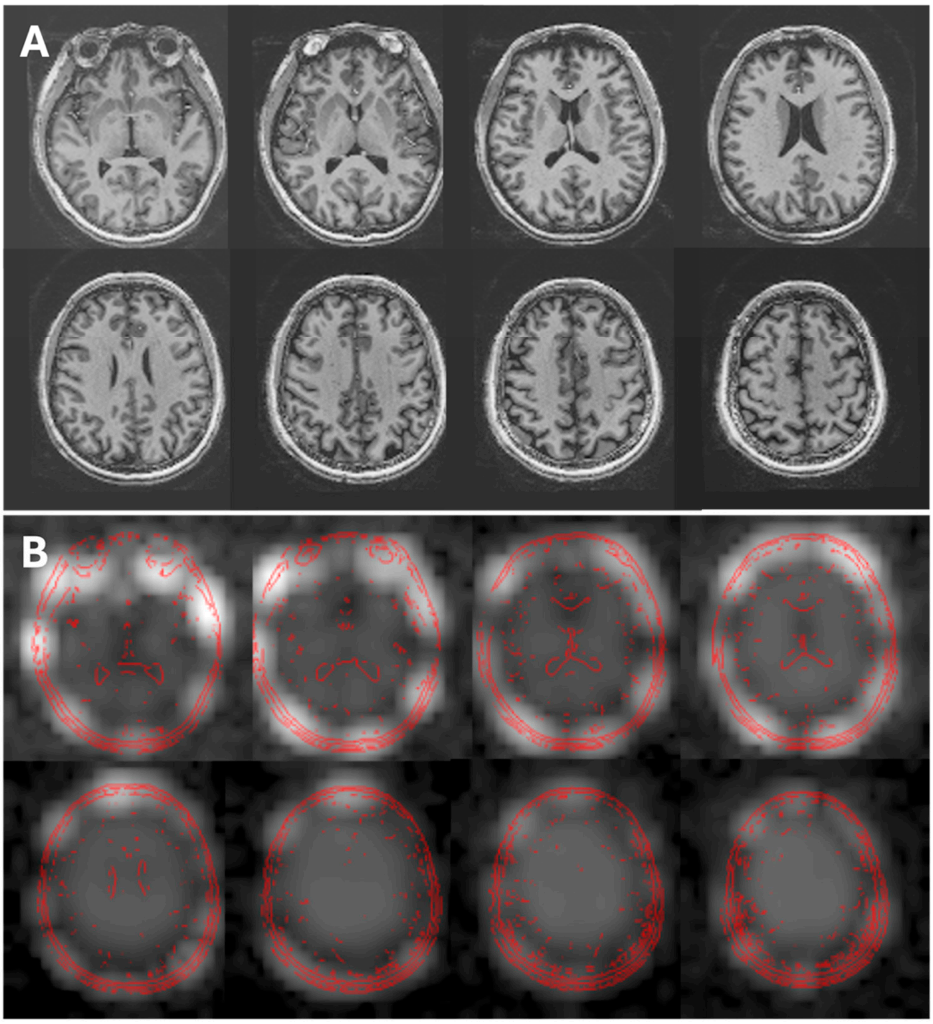

Figure S1. Scouts T1 anatomy (**A**) and magnitude NAA images (**B**) from an eight-slice Hadamard RSI scan, acquired with total scan duration of 17.6 min. The red contours are the edges of the T1 brain images, (**A**) are overlaid on the NAA magnitude images in (**B**).
